# A fast two-stage active contour model for intensity inhomogeneous image segmentation

**DOI:** 10.1371/journal.pone.0214851

**Published:** 2019-04-19

**Authors:** Yangyang Song, Guohua Peng

**Affiliations:** Department of Applied Mathematics, Northwestern Polytechnical University, Xi’an, Shaanxi 710072, PR China; Center for Neuroscience and Regenerative Medicine, UNITED STATES

## Abstract

This paper presents a fast two-stage image segmentation method for intensity inhomogeneous image using an energy function based on a local region-based active contour model with exponential family. In the first stage, we preliminary segment the down-sampled images by the local correntropy-based K-means clustering model with exponential family, which can fast obtain a coarse result with low computational complexity. Subsequently, by taking the up-sampled contour of the first stage as initialization, we precisely segment the original images by the improved local correntropy-based K-means clustering model with exponential family in the second stage. This stage can achieve accurate result rapidly as the result of the proper initialization. Meanwhile, we converge the energy function of two-stage by the Riemannian steepest descent method. Comparing with other statistical numerically methods, which are used to solve the partial differential equations(PDEs), this method can obtain the global minima with less iterations. Moreover, to promote regularity of energy function, we use a popular regular method which is an inner product and applies spatial smoothing to the gradient flow. Extensive experiments on synthetic and real images demonstrate that the proposed method is more efficient than the other state-of-art methods on intensity inhomogeneous images.

## Introduction

Image segmentation is still a popular problem in the field of image processing and computer vision [1]. The intensity inhomogeneity exits in images, which is caused by the imperfections of image acquisition, influence of the illumination and other factors of environment, is one of the main issues in subject of image segmentation. The existences of intensity inhomogeneity in images may lead to misjudgments of doctors and researchers. Therefore, the subject for segmenting images with intensity inhomogeneity attracts more and more researchers to study. Recently, active contour models [2] have been a successful branch for image segmentation.

The existing active contour models can be divided into two major types: edge-based models [3–7] and region-based models [1, 2, 8–15]. Edge-based models utilize the image gradient information to drive the evolution of active contours. Therefore, these models can hardly obtain right segmentation results for images with noisy or weak edge. Alternatively, region-based models use the statistical and curvature information inside and outside of the contour to guide the contour evolution. Hence, the region-based models have better performance than the edge-based models and are able to deal with noisy and blurred images.

One of the most popular region-based models is the Chan-Vese model [2], which computes the intensity averages by using constant intensity information across the region. Thus, this model is a global region-based model and assumes images are homogeneous. For images with intensity inhomogeneity, many local region-based methods [10–15] have been proposed. These models are able to handle image intensity in local region and can be used to segment the images with intensity inhomogeneity. However, most of these region-based models are solved by the standard gradient method [5, 7], which needs a large number of iterations to converge and easily falls into local minima. The convergence problem for active contour models is inherently to solve of the Euler-Lagrange differential equations. Therefore, various methods have been proposed recently to solve the Euler-Lagrange differential equation.

These methods can be mainly categorized into three groups: the first group focuses on investigating more sophisticated differential equations, the second group immerses in searching other alternating direction method, while the third group concentrates on describing Euler’s method as a gradient descent method and applying more advanced optimization techniques. The development of the first group comes from the work of Scheuermann *et al*. [16] and Badshah *et al*. [17]. Scheuermann *et al*. [16] studied the RK-2 and RK-3 order Runge-Kutta methods and the explicit Euler method. Badshah *et al*. [17] proposed two related multigrid algorithm. The second group derives from Xie *et al*. [18] and Song *et al*. [19]. Xie *et al*. [18] and Song *et al*. [19] considered Bregman method and Split Bregman method for image segmentation. These methods offered another line of fast algorithms and can get global minima for some active contour models. But the Bregman method and Split Bregman method are used to solve *L*_0_ minimization directly and do not work for different types active contour models.

As far as the optimization-based approach, Chartrand *et al*. [20] and Mendi *et al*. [21] proposed the quasi-Newton method for minimizing the active contour energy function within Chan-Vese model. Following these two works, Bar *et al*. [22] modified the quasi-Newton method by adding an analytic functional Hessian and presented a generalized Newton method. Alternatively, Sundaramoorthi *et al*. [23] creatively proposed the Sobelev gradient descent method by using Sobolev metrics. The Sobelev metrics outperform the traditional metrics for the same segmentation energy function.

Motivated by the work of Sundaramoorthi *et al*. [23], Pereyra *et al*. [24] first considered the Riemannian steepest descent method on model’s intrinsic manifold for the Chan-Vese model, which converged extremely fast. However, this method can only be applied to images with Gaussian distribution. Considering that the images have other specific distributions, such as Gamma [25], Rayleigh [26], Laplace [10] and Weibull [27]. Pereyra *et al*. [28] derived a general Riemannian optimization method for active contour model with the exponential family [29]. More precisely, Pereyra *et al*. presented a smooth descent algorithm [30] for nonparametric active contour models. Above all, Pereyra *et al*. have given appropriate mathematical development for natural metric of the statistical manifold, which makes the computation of natural matric possible. But in the paper [28], Pereyra *et al*. only demonstrated the feasibility of the natural metric for Chan-Vese model, which still cannot achieve satisfactory segmentation results for images with intensity inhomogeneity.

To achieve the object of fast segmentation for images with intensity inhomogeneity, we combine the Riemannian steepest descent method with local correntropy-based K-means clustering (LCK) model [13], which could obtain ideal segmentation results for intensity inhomogeneous images. In addition, in order to further improve the speed of operation and weaken the dependence of LCK model on the initial position, we divide our segmentation algorithm into two stages. The first stage can roughly but fast obtain the coarse contour near the object boundaries with low computational complexity in coarse space. The second stage can easily get the accurate segmentation results with suitable initialization in original space. Meanwhile, the two minimization problems of the energy function with exponential family [29] are iterated by natural gradient method, which is faster than other methods for solving active contour models to find the global minima.

The remainder of this paper is structured as follows. In the next section we introduce the classic region-based active contour modes and the frameworks of region-based active contour models with exponential family. In Section 3 we derive a fast two-stage segmentation model. More precisely, we develop the mathematical implement for local region-based active contour models. In Section 4 we list the experimental results illustrating the performance of the proposed method. Finally, in Section 5 we describe the conclusion and future work.

## Background and theoretical foundations

### Traditional region-based active contour models

#### Chan-Vese model

Chan and Vese [2] proposed a global region-based active contour model for image segmentation. This model approximates the image intensities outside and inside of contour by the average image intensities outside and inside of contour, respectively. For the input image *I*: Ω → ℜ^2^, we divide the image domain Ω into two regions: an object region Ω_*inside*_ and a background region Ω_*outside*_ = Ω/Ω_*inside*_ by a closed contour *C*(*s*): [0, 1] → ℜ^2^, which corresponding to zero level set function: *C* = {*x*: *ϕ*(*x*) = 0}. The energy function of Chan-Vese model can be written as:

$$\begin{array}{ccc} E_{CV}\left(C,c_{1},c_{2}\right)= & & \lambda_{1}\int_{\Omega }{\left|I\left(x\right)-c_{1}\right|}^{2}H_{\epsilon }\left(\phi \left(x\right)\right)\;dx\\ & & +\lambda_{2}\int_{\Omega }{\left|I\left(x\right)-c_{2}\right|}^{2}\left(1-H_{\epsilon }\left(\phi \left(x\right)\right)\right)\;dx\\ & & +\mu \int_{\Omega }{\left|\nabla H_{\epsilon }\left(\phi \left(x\right)\right)\right|}^{2}dx+\nu \int_{\Omega }H_{\epsilon }\left(x\right)\;dx. \end{array}$$ (1)

Where *μ* ≥ 0, *ν* ≥ 0, λ_1_ ≥ 0 and λ_2_ ≥ 0 are fixed constants. *c*_1_ and *c*_2_ are the average image intensities outside and inside contour *C*, respectively. *H*_*ϵ*_(*ϕ*) is the smooth approximate of the Heaviside function

$$\begin{array}{c} H_{\epsilon }\left(x\right)=\frac{1}{2}\left[1+\frac{1}{\pi }\text{arctan}\left(\frac{x}{\epsilon }\right)\right] \end{array}$$ (2)

and the derivative of *H*_*ϵ*_ is defined as

$$\begin{array}{c} \delta_{\epsilon }\left(x\right)=H_{\epsilon }^{{}^{\prime }}\left(x\right)=\frac{\epsilon }{\pi (\epsilon^{2}+x^{2})}. \end{array}$$ (3)

Chan and Vese minimized the energy function (1) by the standard gradient descent method [31]. The gradient descent flow corresponding to *ϕ* can be written as

$$\begin{array}{c} \left\{ \begin{array}{c} c_{1}\left(x\right)=\frac{\int_{\Omega }I\left(x\right)H_{\epsilon }\left(\phi \left(x\right)\right)\;dx}{\int_{\Omega }H_{\epsilon }\left(\phi \left(x\right)\right)\;dx},\;c_{2}\left(x\right)=\frac{\int_{\Omega }I\left(x\right)\left(1-H_{\epsilon }\left(\phi \left(x\right)\right)\right)\;dx}{\int_{\Omega }\left(1-H_{\epsilon }\left(\phi \left(x\right)\right)\right)\;dx}\\ \frac{\partial \phi }{\partial t}=\delta_{\epsilon }\left(\phi \right)\left(-\lambda_{1}{\left(I\left(x\right)-c_{1}\right)}^{2}+\lambda_{2}{\left(I\left(x\right)-c_{2}\right)}^{2}+\mu \text{div}\left(\frac{\nabla \phi }{\left|\nabla \phi \right|}\right)-\nu \right) \end{array}.\right. \end{array}$$ (4)

As a global active contour model, the curve convolution of the CV model is only related to global characteristic of the image region. Therefore, the CV model can achieve the satisfactory results for images with intensity homogeneity, whereas it can not deal with the images with intensity inhomogeneity.

#### Local binary fitted model

Li *et al*. [11] proposed the local binary fitting (LBF) model by considering a kernel function into a local region-based model. The LBF model can deal with intensity inhomogeneity images. The energy function of LBF model is defined as

$$\begin{array}{ccc} E_{LBF}\left(\phi \right)= & & -\lambda_{1}\int_{\Omega }\int_{\Omega }K_{\sigma }\left(x-y\right){\left|I\left(y\right)-m_{1}\left(x\right)\right|}^{2}H\left(\phi \left(y\right)\right)\;dy\;dx\\ & & -\lambda_{2}\int_{\Omega }\int_{\Omega }K_{\sigma }\left(x-y\right){\left|I\left(y\right)-m_{2}\left(x\right)\right|}^{2}\left(1-H\left(\phi \left(y\right)\right)\right)\;dy\;dx\\ & & +\nu \int_{\Omega }|\nabla H\left(\phi \left(x\right)\right)|\;dx+\mu \int_{\Omega }\frac{1}{2}{\left(|\nabla \phi \left(x\right)|-1\right)}^{2}\;dx, \end{array}$$ (5)

where λ_1_, λ_2_ ≥ 0, *ν* ≥ 0 and *μ* ≥ 0 are fixed constants. $K_{\sigma }\left(x-y\right)=\frac{1}{{\left(2\pi \right)}^{\left(n/2\right)}\sigma^{n}}$ $e^{{-|x-y|}^{2}/2\sigma^{2}}$ is Gaussian kernel function with kernel width *σ*, which is introduced to control the spatial distance between the *x*-th and the *y*-th; *m*_1_(*x*) and *m*_2_(*x*) are the local clusters of the *x*-th pixel. Meanwhile, in order to regularize the level set function *ϕ*, the third and the forth terms are added as level set regularization term.

The level set function and the local clusters can be updated from Eq (5) by the standard gradient descent method, which can be computed as

$$\begin{array}{c} \left\{ \begin{array}{c} m_{1}\left(x\right)=\frac{K_{\sigma }\left(x\right)*\left[H\left(\phi \left(x\right)\right)I\left(x\right)\right]}{K_{\sigma }\left(x\right)*H\left(\phi \left(x\right)\right)},\;m_{2}\left(x\right)=\frac{K_{\sigma }\left(x\right)*\left[\left(1-H\left(\phi \left(x\right)\right)\right)I\left(x\right)\right]}{K_{\sigma }\left(x\right)*\left(1-H\left(\phi \left(x\right)\right)\right)}\\ \frac{\partial \varphi }{\partial t}=-\delta_{\epsilon }\left(\phi \right)\left(\lambda_{1}e_{1}-\lambda_{2}e_{2}\right)+\nu \delta_{\epsilon }\left(\phi \right)\text{div}\left(\frac{\nabla \phi }{\left|\nabla \phi \right|}\right)+\mu \left(\nabla^{2}\phi -\text{div}\left(\frac{\nabla \phi }{\left|\nabla \phi \right|}\right)\right). \end{array}\right. \end{array}$$ (6)

Where *e*_1_ and *e*_2_ are

$$\begin{array}{c} e_{i}=\int_{\Omega }K_{\sigma }\left(y-x\right){\left|I\left(x\right)-m_{i}\left(y\right)\right|}^{2}\;dy.\;i=1,2 \end{array}$$

The LBF model considers the Gaussian kernel function to capture the local region information of images and can segment the images with intensity inhomogeneity. However, the Gaussian kernel function is not sufficient to introduce the image information and the LBF model cannot segment images with severe intensity inhomogeneity. Moreover, this model is very sensitive to the initialization and the iterative approach of it easily falls into local minima.

#### Local correntropy-based K-means clustering model

Wang *et al*. [13] presented a more accurate segmentation method (LCK) for images with unknown complex noise. The main difference between the LCK model and LBF model is that the LCK model utilizes the pixel-to-cluster distance, which makes this model is more robust to unknown complex noise. The objective function of the LCK model is

$$\begin{array}{ccc} E_{LCK}\left(\phi \right)= & & -\lambda_{1}\int_{\Omega }\int_{\Omega }K_{\sigma }\left(x-y\right)w_{y}{\left|I\left(y\right)-m_{1}\left(x\right)\right|}^{2}H\left(\phi \left(y\right)\right)\;dy\;dx\\ & & -\lambda_{2}\int_{\Omega }\int_{\Omega }K_{\sigma }\left(x-y\right)w_{y}{\left|I\left(y\right)-m_{2}\left(x\right)\right|}^{2}\left(1-H\left(\phi \left(y\right)\right)\right)\;dy\;dx\\ & & +\nu \int_{\Omega }|\nabla H\left(\phi \left(x\right)\right)|\;dx+\mu \int_{\Omega }\frac{1}{2}{\left(|\nabla \phi \left(x\right)|-1\right)}^{2}\;dx. \end{array}$$ (7)

The weight of the *y*-th is calculated by

$$\begin{array}{c} w_{y}=\int_{\Omega }H\left(\phi \left(x\right)\right)g(\|I\left(y\right)-m_{1}{\left(x\right)\|}_{2})+\left(1-H\left(\phi \left(x\right)\right)\right)g(\|I\left(y\right)-m_{2}\left(x\right){\|}_{2}). \end{array}$$ (9)

Minimizing Eq (7) by using the standard gradient method, the corresponding local clusters and level set formulation is obtained by

$$\begin{array}{c} \left\{ \begin{array}{c} m_{1}\left(x\right)=\frac{K_{\sigma }\left(x\right)*\left[H\left(\phi \left(x\right)\right)I\left(x\right)\right]w_{x}}{K_{\sigma }\left(x\right)*H\left(\phi \left(x\right)\right)w_{x}},\;m_{2}\left(x\right)=\frac{K_{\sigma }\left(x\right)*\left[\left(1-H\left(\phi \left(x\right)\right)\right)I\left(x\right)\right]w_{x}}{K_{\sigma }\left(x\right)*\left(1-H\left(\phi \left(x\right)\right)\right)w_{x}}\\ \frac{\partial \varphi }{\partial t}=-H\left(\phi \right)\delta_{\epsilon }\left(\phi \right)w_{x}\left(\lambda_{1}e_{1}-\lambda_{2}e_{2}\right)+\nu \delta_{\epsilon }\left(\phi \right)\text{div}\left(\frac{\nabla \phi }{\left|\nabla \phi \right|}\right)+\mu \left(\nabla^{2}\phi -\text{div}\left(\frac{\nabla \phi }{\left|\nabla \phi \right|}\right)\right). \end{array}\right. \end{array}$$ (8)

The LCK model is robust to images with complex noise and intensity inhomogeneity, whereas it is also a little bit sensitive to the initialization. Meanwhile, the iterative method of the LCK model also easily falls into minima and needs much more times to obtain the final segmentation results.

### Region-based model with exponential family observation

Due to the fact that the energy functions of region-based active contour model does not have a structure of a vector space, Lecellier *et al*. [29] proposed the exponential family observation framework for region-based model. This framework is specifically calculated when coping with global region-based information such as statistical image features (histogram, variance and mean).

#### Exponential family

In this section, we provide the necessary mathematical concepts on the exponential family, which covers most noise models of the image acquisition system. For the given point *x* ∈ ℜ^*d*^, the image values can be distributed by

$$\begin{array}{c} I\left(x\right)∼f\left(\cdot |m_{1}\left(x\right)\right)\;if\;x\in \Omega_{inside},\;I\left(x\right)∼f\left(\cdot |m_{2}\left(x\right)\right)\;if\;x\in \Omega_{outside}. \end{array}$$ (9)

Where the *m*_1_(*x*) and *m*_2_(*x*) are the clusters of the foreground and background respectively and the function *f*: ℜ^*p*^ → ℜ^+^ is the probability density function with exponential family distribution.

**Definition 0.1** *The family of distributions of a random variable, is called a k-parameter canonical exponential family. If there exists canonical parameter vector η* = (*η*_1_, …, *η*_*k*_)^*T*^ *and log-normalizer A*(*η*), *and real-valued functions h*, *T*_1_, …, *T*_*k*_: ℜ^*k*^ → ℜ, *the probability density function f*(⋅|*m*) *may be written as*

$$\begin{array}{c} f\left(s|m\right)=h\left(y\right)\text{exp}[\eta {\left(m\right)}^{T}T\left(y\right)-A\left(m\right)], \end{array}$$ (10)

*here T* = (*T*_1_, …, *T*_*k*_)^*T*^ *is the sufficient statistic*.

Note the Definition 0.1 gives the general distribution of exponential family that most of them have distribution for signal and image processing. Table 1 shows some common canonical distributions of the exponential family, such as the Gamma, Beta, Poisson, Gaussian, Exponential, and Rayleigh.

**Table 1:** Some common distribution of the exponential family.

Distribution	*m*^*T*^	*η*(*m*)	S(s)	A(m)
Gamma	(λ, *p*)	(−λ, *p* − 1)	(*s*, log*s*)	−(*η*_2_ + 1)log − *η*_1_ + logΓ(*η*_2_ + 1)
Beta	(r,s)	(r-1,s-1)	(log*s*, log(1 − *s*))	−log*B*(*η*_1_ + 1, *η*_2_ + 1)
Poisson	*μ*	log*μ*	s	*e*^*η*^
Gaussian	(*μ*, *σ*^2^)	$\left(\frac{\mu }{\sigma^{2}},\frac{-1}{-2\sigma^{2}}\right)$	(*s*, *s*^2^)	$\frac{1}{2}\left(-\frac{\eta_{1}^{2}}{2\eta_{2}}-\text{log}\frac{-\eta_{2}}{\pi }\right)$
Exponential	λ	−λ	s	−log − *η*
Rayleigh	*m*^2^	$-\frac{1}{2m^{2}}$	*s*^2^	−log − 2*η*

#### Region-based active contour model with exponential family observation

Following the definition of exponential family, the region-based active contour model (this paper takes LCK model as an example)(Eq (7)) with exponential family observation can be rewritten as

$$\begin{array}{ccc} E_{LCK}\left(\phi \right)= & & -\lambda_{1}\int_{\Omega }\int_{\Omega }K_{\sigma }\left(x-y\right)w_{y}\;\text{log}\;f\left[I\left(y\right)|m_{1}\left(x\right)\right]H\left(\phi \left(y\right)\right)\;dy\;dx\\ & & -\lambda_{2}\int_{\Omega }\int_{\Omega }K_{\sigma }\left(x-y\right)w_{y}\;\text{log}\;f\left[I\left(y\right)|m_{2}\left(x\right)\right]\left(1-H\left(\phi \left(y\right)\right)\right)\;dy\;dx. \end{array}$$ (11)

The functional optimization problem Eq (12) can be solved by alternative minimization method. In this paper, we take the Gaussian distribution as an example and give the iteration with respect to *ϕ*, *m*_1_(*x*) and *m*_2_(*x*) as following:

$$\begin{array}{c} \left\{ \begin{array}{c} m_{1}\left(x\right)=\frac{K_{\sigma }\left(x\right)*\left[H\left(\phi \left(x\right)\right)I\left(x\right)\right]w_{x}}{K_{\sigma }\left(x\right)*H\left(\phi \left(x\right)\right)w_{x}},\;m_{2}\left(x\right)=\frac{K_{\sigma }\left(x\right)*\left[\left(1-H\left(\phi \left(x\right)\right)\right)I\left(x\right)\right]w_{x}}{K_{\sigma }\left(x\right)*\left(1-H\left(\phi \left(x\right)\right)\right)w_{x}}\\ \frac{\partial \phi }{\partial t}=K_{\sigma }*\left\{\delta \left(\phi \left(x\right)\right)w_{x}\left[\lambda_{1}\;\text{log}\;f\left(I\left(x\right)|m_{1}\left(x\right)\right)-\lambda_{2}\;\text{log}\;f\left(I\left(x\right)|m_{2}\left(x\right)\right)\right]\right\}. \end{array}\right. \end{array}$$ (12)

Although we give more general distribution for LCK model, it still can not overcome its inherent limitations. The segmentation method of the above LCK models is not convex and the weights of the pixels *w*_*x*_ are always decreased faster than other region pixels during the iterated process. Hence, the main difficulty for the region-based active contour model is still the solution of energy function.

## Proposed model

Inspired by the work of Wang *et al*. [13] and Pereyra *et al*. [28], we proposed a novel two-stage segmentation method, which can fast segment the intensity inhomogeneous images with accuracy results. In the real-world, the accuracy segmentation results for intensity inhomogeneity images are difficult to obtain and also need to spend a large amount of time, because these intensity inhomogeneity images are always big and complex. Due to the above factor, we split the process of the segmentation into two stages which makes the computation with lower computational complexity. In the first stage, we implement the segmentation model in coarse spaces. Following it, in the accurate segmentation stage, we utilize the up-sampled coarse results as initializations and segment the images in fine space. Finally, to get more accurate segmentation results faster, we present the Riemannian steepest descent method for the two-stage segmentation model.

### Coarse segmentation

The LCK model is robust to images with severe intensity inhomogeneity and complex noise. However, as a local region-based active contour, this model needs a perfect initialization to obtain the satisfactory segmentation results. In the first stage, we propose a novel LCK model with exponential family observation in coarse space (RDLCK), which can obtain the segmentation results fast. The energy functional of this model can be defined as

$$\begin{array}{ccc} & & E^{RDLCK}\left(\phi_{\tau }\right)=\\ & & -\lambda_{1}\int_{\Omega_{\tau }}\int_{\Omega_{\tau }}K_{\sigma }\left(x-y\right)w_{\tau y}\;\text{log}\;f\left[I\left(y\right)|m_{\tau 1}\left(x\right)\right]H\left(\phi_{\tau }\left(y\right)\right)\;dy\;dx\\ & & -\lambda_{2}\int_{\Omega_{\tau }}\int_{\Omega_{\tau }}K_{\sigma }\left(x-y\right)w_{\tau y}\;\text{log}\;f\left[I\left(y\right)|m_{\tau 2}\left(x\right)\right]\left(1-H\left(\phi_{\tau }\left(y\right)\right)\right)\;dy\;dx. \end{array}$$ (13)

Where Ω_*τ*_ denotes the uniform sub-space of Ω with the down-sampled factor *τ*, and function *f* is exponential family function. *w*_*τy*_ is computed by

$$\begin{array}{c} w_{\tau y}=\int_{\Omega_{\tau }}H\left(\phi_{t}\left(y\right)\right)g(\|I\left(y\right)-m_{\tau 1}{\left(x\right)\|}_{2})+\left(1-H\left(\phi_{t}\left(y\right)\right)\right)g(\|I\left(y\right)-m_{\tau 2}\left(x\right){\|}_{2})\;dy, \end{array}$$

and *g*(*x*) is Gaussian kernel function *g*(*x*) = exp(−*x*^2^/2*σ*^2^). *m*_*τ*1_(*x*) and *m*_*τ*2_(*x*) are the local clusters in the coarse space, which can be calculated by gradient descent method. For *f* is Gaussian distribution, then *m*_*τ*1_(*x*) and *m*_*τ*2_(*x*) can be computed by

$$\begin{array}{c} m_{\tau 1}\left(x\right)=\frac{K_{\sigma }\left(x\right)*\left[H\left(\phi_{\tau }\left(x\right)\right)I\left(x\right)\right]w_{\tau x}}{K_{\sigma }\left(x\right)*H\left(\phi_{\tau }\left(x\right)\right)w_{\tau x}},\\ m_{\tau 2}\left(x\right)=\frac{K_{\sigma }\left(x\right)*\left[\left(1-H\left(\phi_{\tau }\left(x\right)\right)\right)I\left(x\right)\right]w_{\tau x}}{K_{\sigma }\left(x\right)*\left(1-H\left(\phi_{\tau }\left(x\right)\right)\right)w_{\tau x}}. \end{array}$$ (14)

Most traditional region-based active contour models always converged by using the standard gradient descent method, whereas it easily falls into minima and needs a large number of iterations. Therefore, fixing *m*_*τ*1_(*x*) and *m*_*τ*2_(*x*), we utilize the Riemannian steepest descent method for RDLCK model. The update for level set function *ϕ*_*τ*_ is

$$\begin{array}{c} \phi_{\tau }^{t+1}=\phi_{\tau }^{t}-\gamma_{\tau t}{N_{\tau }}^{-1}\left(\phi_{\tau }^{t}\right)\nabla_{\phi_{\tau }}E^{RDLCK}\left(I\left(x\right);\phi_{\tau }^{t}\right). \end{array}$$ (15)

Here *γ*_*τt*_ is a positive parameter, $\nabla_{\phi_{\tau }}E^{RDLCK}\left(I\left(x\right);\phi_{\tau }^{t}\right)$ is the standard gradient flow, which is similar to Eq (12)

$$\begin{array}{ccc} \nabla_{\phi_{\tau }}E^{RDLCK}\left(I\left(x\right);\phi_{\tau }^{t}\right)= & & K_{\sigma }*\{\delta \left(\phi_{\tau }\left(x\right)\right)w_{\tau x}[\lambda_{1}\;\text{log}\;f\left(I\left(x\right)|m_{\tau 1}\left(x\right)\right)\\ & & -\lambda_{2}\;\text{log}\;f\left(I\left(x\right)|m_{\tau 2}\left(x\right)\right)]\} \end{array}$$ (16)

and ${N_{\tau }}^{-1}\left(\phi_{\tau }^{t}\right)\nabla_{\phi_{\tau }}E^{RDLCK}\left(I\left(x\right);\phi_{\tau }^{t}\right)$ is the Riemannian steepest descent of *E*^*RDLCK*^ on Euclidean tangent space [30] and $N_{\tau }\left(\phi_{\tau }\right)$ is

$$\begin{array}{c} {\left(N_{\tau }\left(\phi_{\tau }\right)\right)}_{\left(x,y\right)}=K_{\sigma }\left(x\right)*\{\begin{array}{c} \{|\delta \left(\phi_{\tau }\left(x\right)\right)|w_{\tau x}B_{f}(m_{\tau 2}\left(x\right)||m_{\tau 1}\left(x\right))\}\;if\;\phi_{\tau }\left(x\right)\geq 0\\ \{|\delta \left(\phi_{\tau }\left(x\right)\right)|w_{\tau x}B_{f}(m_{\tau 1}\left(x\right)||m_{\tau 2}\left(x\right))\}\;if\;\phi_{\tau }\left(x\right)\leq 0 \end{array}. \end{array}$$ (17)

Therefore, the key to Eq (16) is that the $N_{\tau }$ is a positive definite matrix. (refer to the appendix for a detailed computation for N).

This paper makes the computation of the ${N_{\tau }}^{-1}\left(\phi_{\tau }^{t}\right)\nabla_{\phi_{\tau }}E^{RDLCK}\left(y;\phi_{\tau }^{t}\right)$ possible. Moreover, the fast image segmentation method based on natural gradient Eq (15) is applied to local region-based active contour model successfully.

Lastly, to promote the solutions of Eq (13) smooth, we need to regularise *ϕ*. The traditional region-based active contour model is regularised by adding the penalty term $\lambda \int_{\Omega }|\delta \left[\phi \left(x\right)\right]|\;dx$, which regularises the energy function by selecting contours with minimal length. In this paper, we promote regularity by using an increasingly popular alternative, which applies spatial smoothing to the natural gradient flow. This method can be written as

$$\begin{array}{c} \phi_{\tau }^{t+1}=\phi_{\tau }^{t}+\gamma_{\tau t}H_{\sigma }N^{-1}\phi_{\tau }^{t}\nabla E_{\epsilon }^{RDLCK}\left(I\left(x\right);\phi_{\tau }^{t}\right). \end{array}$$ (18)

Where $h_{\sigma }\left(s,u\right)=\frac{1}{2\pi \sigma^{2}}\text{exp}\left(-\frac{s^{2}+u^{2}}{2\sigma^{2}}\right)$ and the low values of *σ* are applied for images with low noise, which preserves details of images.

**Fig 1:**
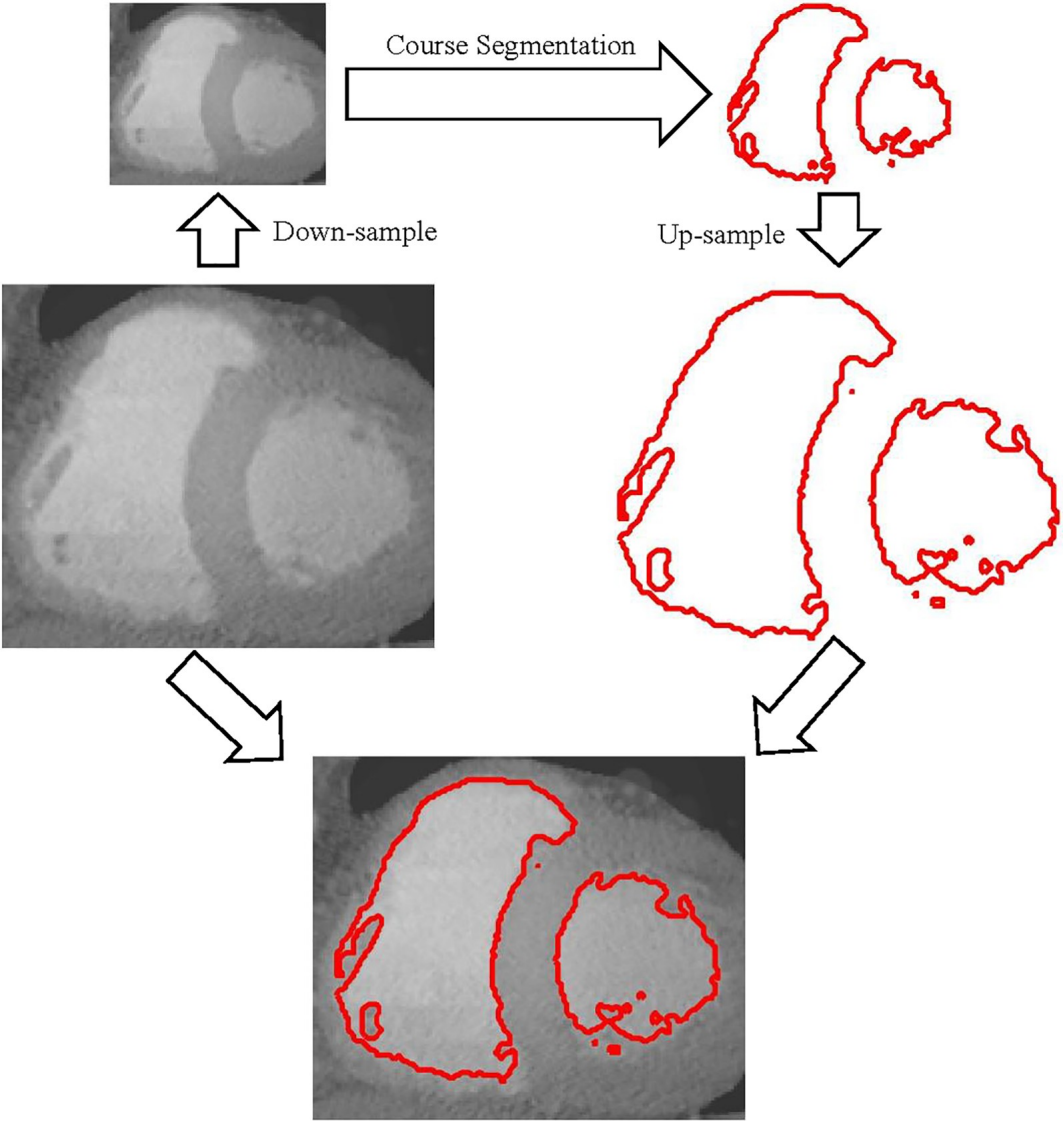
The process of obtaining the initialization for accurate segmentation.

We can obtain the coarse contour *ϕ*_*τ*_ in the down-sampled space Ω_*τ*_, but the coarse contour *ϕ*_*τ*_ is not suitable for the original image due to down-sampling process. Therefore, we need to up-sample the coarse contour *ϕ*_*τ*_ and get the up-sampling version ϕ* of the coarse contour *ϕ*_*τ*_. As shown in Fig 1, we give the segmentation process of a heart CT image (the size of the image is 152 × 128).

### Accurate segmentation

The coarse segmentation can obtain the coarse contour with low cost of computation, whereas the coarse contour is not accurate due to the loss of region information from down-sampling. Therefore, we further segment the objects by improved LCK model with satisfactory initialization ϕ*. The improved LCK model with exponential family in original space (ROLCK) is defined as

$$\begin{array}{ccc} E^{ROLCK}\left(\phi \right)= & & -\lambda_{1}\int_{\Omega }\int_{\Omega }K_{\sigma }\left(x-y\right)w_{y}\;\text{log}\;f\left[I\left(y\right)|m_{1}\left(x\right)\right]H\left(\phi \left(y\right)\right)\;dy\;dx\\ & & -\lambda_{2}\int_{\Omega }\int_{\Omega }K_{\sigma }\left(x-y\right)w_{y}\;\text{log}\;f\left[I\left(y\right)|m_{2}\left(x\right)\right]\left(1-H\left(\phi_{(}y\right)\right))\;dy\;dx\\ & & -\xi \int_{\Omega }\;\text{log}\;f\left[\phi \left(x\right)|\phi^{*}\left(x\right)\right]\;dx, \end{array}$$ (19)

where *ξ* is a small positive parameter and ϕ* is the up-sampling vision of the coarse contour. The last term promise that the distance between ϕ* and accurate result is small.

Using the Riemannian steepest descent method for converging ROLCK model, the iterative scheme for minimizing the Eq (19) is similar with the one for solving Eq (13).

$$\begin{array}{c} \left\{ \begin{array}{c} m_{1}\left(x\right)=\frac{K_{\sigma }\left(x\right)*\left[H\left(\phi_{\tau }\left(x\right)\right)I\left(x\right)\right]w_{x}}{K_{\sigma }\left(x\right)*H\left(\phi_{(}x\right))w_{x}},\;m_{2}\left(x\right)=\frac{K_{\sigma }\left(x\right)*\left[\left(1-H\left(\phi_{(}x\right)\right)\right)I\left(x\right)]w_{x}}{K_{\sigma }\left(x\right)*\left(1-H\left(\phi_{(}x\right)\right))w_{x}}\\ \phi^{t+1}=\phi^{t}+\gamma_{t}H_{\sigma }N^{-1}\phi^{t}\nabla E_{\epsilon }^{ROLCK}\left(I\left(x\right);\phi^{t}\right). \end{array}\right. \end{array}$$ (20)

Where *γ*_*t*_ is positive constant and *w*_*x*_ is the final weight of the *x*-th pixel, which is calculated by *w*_*x*_ = ∫_Ω_ *H*(*ϕ*(*y*))*g*(‖*I*(*x*) − *m*_1_(*y*)‖^2^) + (1 − *H*(*ϕ*(*x*)))*g*(‖*I*(*x*) − *m*_2_(*y*)‖^2^), the computation of N is also similar to the computation of $N_{\tau }$

$$\begin{array}{c} {\left(N\left(\phi \right)\right)}_{\left(x,y\right)}=K_{\sigma }\left(x\right)*\{\begin{array}{c} \left\{|\delta \left(\phi \left(x\right)\right)\right|w_{x}B_{f}(m_{2}\left(x\right)||m_{1}\left(x\right))\}\;if\;\phi \left(x\right)\geq 0\\ \left\{|\delta \left(\phi \left(x\right)\right)\right|w_{x}B_{f}(m_{1}\left(x\right)||m_{2}\left(x\right))\}\;if\;\phi \left(x\right)\leq 0 \end{array}. \end{array}$$ (21)

The only difference is the iteration of $\nabla_{\phi }E_{\epsilon }^{ROLCK}\left(I\left(x\right);\phi^{t}\right)$, it is computed by

$$\begin{array}{ccc} \nabla_{\phi }E^{ROLCK}\left(I\left(x\right);\phi^{t}\right)= & & K_{\sigma }*\{\delta \left(\phi \left(x\right)\right)w_{x}[\lambda_{1}\;\text{log}\;f\left(I\left(x\right)|m_{1}\left(x\right)\right)\\ & & -\lambda_{2}\;\text{log}\;f\left(I\left(x\right)|m_{2}\left(x\right)\right)]\}+2\xi |\phi -\phi^{*}|. \end{array}$$ (22)

At the original space, there is no region information loss. Since the initialization for Eq (19) is very close to the object, the convergence of Eq (19) is fast and the accurate segmentation stage can obtain the final result after a few iterations.

### Segmentation procedures

The procedures of the proposed method can be summarized in Algorithm. 1. The initial level function of the coarse segmentation is defined as

$$\begin{array}{c} \phi_{\tau }^{0}\left(y\right)=\{\begin{array}{c} \rho \;y\in \text{rectangle}\;\text{inner}\\ 0\;y\in \text{rectangle}\;\text{boundary}\\ -\rho \;y\in \text{rectangle}\;\text{outer} \end{array}. \end{array}$$ (23)

We provide a positive parameter for *ρ* in this paper.

The proposed method can be summarized in Algorithm 1.

## Experimental results and comparison

In order to prove and compare the performance with the CV model [2], the GCV model [10], the LBF model [11], the LIC model [12], the LCK model [13], the RCV model [28] and the LGFI model [14], we apply our approach to several images with intensity inhomogeneity in this section. All the experiments are implemented by using MATLAB R2015a and running on a person computer with Intel core i5, 2.5GHz, and 4.00 GB RAM.

**Algorithm 1** Two-stage Segmentation

**Stage 1**: Coarse Segmentation on Ω_*τ*_

1: **Input**: Ω_*τ*_

2: **Initialization**: $\phi_{\tau }^{0}$ by Eq (23)

3: **for** 1 to MaxIter **do**

4:  Compute

5:  Clusters *m*_*τ*1_(*x*) and *m*_*τ*2_(*x*) by Eq (14)

6:  $\nabla_{\phi_{\tau }}E^{RDLCK}\left(I\left(x\right);\phi_{\tau }^{t}\right)$ by Eq (16)

7:  $N_{\tau }\left(\phi_{\tau }\right)$ by Eq (17)

8:  Updating the level set function *ϕ*_*τ*_ by Eq (15)

9: **end for**

10: **Output**: Coarse segmentation result ${\hat{\phi }}_{\tau }$,

**Stage 2**: Accurate Segmentation on Ω

11: **Let** ϕ* be the up-sampling version of ${\hat{\phi }}_{\tau }$,

12: **for** 1 to MaxIter **do**

13:  Compute

14:  Clusters *m*_1_(*x*) and *m*_2_(*x*) by Eq (20)

15:  ∇_*ϕ*_ *E*^*ROLCK*^(*I*(*x*); *ϕ*^*t*^) by Eq (22)

16:  N(ϕ) by Eq (21)

17:  Updating the level set function *ϕ* by Eq (20)

18: **end for**

### Comparisons with state-of-art methods for intensity inhomogeneity images

#### Comparisons with global active contour models (the CV [2] model, GCV [10] model and RCV [28] model

In this section, we compare our method with some global active contour models. The global active contour models assume that the intensities of images are piecewise constant and thereby these models can not segment the images with intensity inhomogeneity.

**Fig 2:**
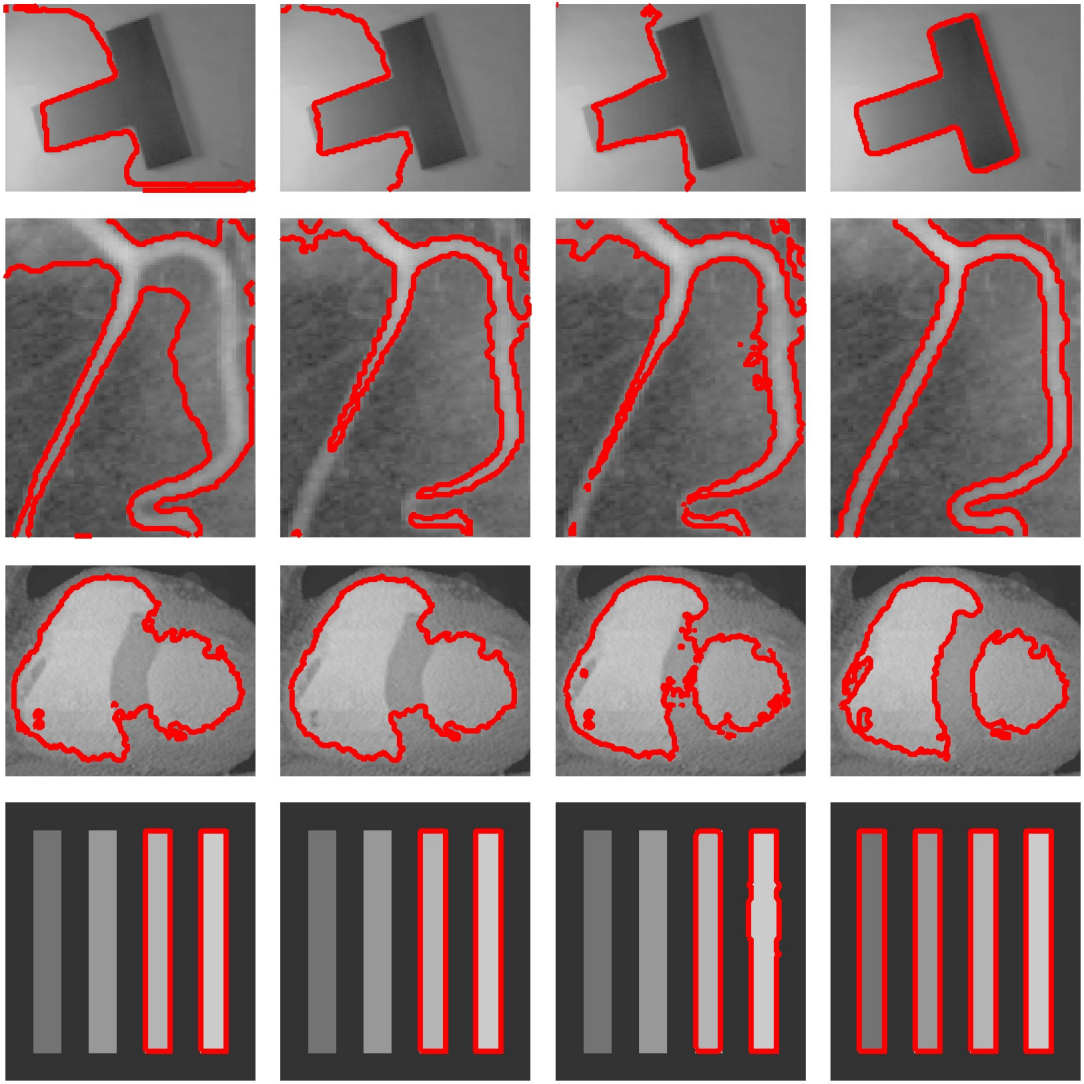
Comparisons with global active contour models using two synthetic images and two medical images. From left to right: segmentation results by the CV model, the GCV model and the RCV model.

Fig 2 shows the segmentation results on two synthetic images (The first row is a T-shape image and the fourth row is a synthetic image.) and two medical images (The second row is a blood image and the third row is a CT heart image.) with intensity inhomogeneity by using the CV model [2], the GCV model [10], the RCV model [28] and the proposed method, respectively. The first column shows the segmentation results with CV model. The second column shows the segmentation results with GCV model. The third column shows the segmentation results with RCV model. Finally the last column shows the segmentation results with our method. It can be seen that the results of the CV model [2], the GCV model [10] and the RCV model [28] are unsatisfactory because these models are global active contour models and use the global intensity means of images to fit them. On the other hand, our method can achieve satisfying results due to taking the local image region information into account, which can deal with the intensity inhomogeneous images well.

#### Comparisons with local active contour models (the LIC [12] model, the LCK [13] model and the LGFI model [14])

**Fig 3:**
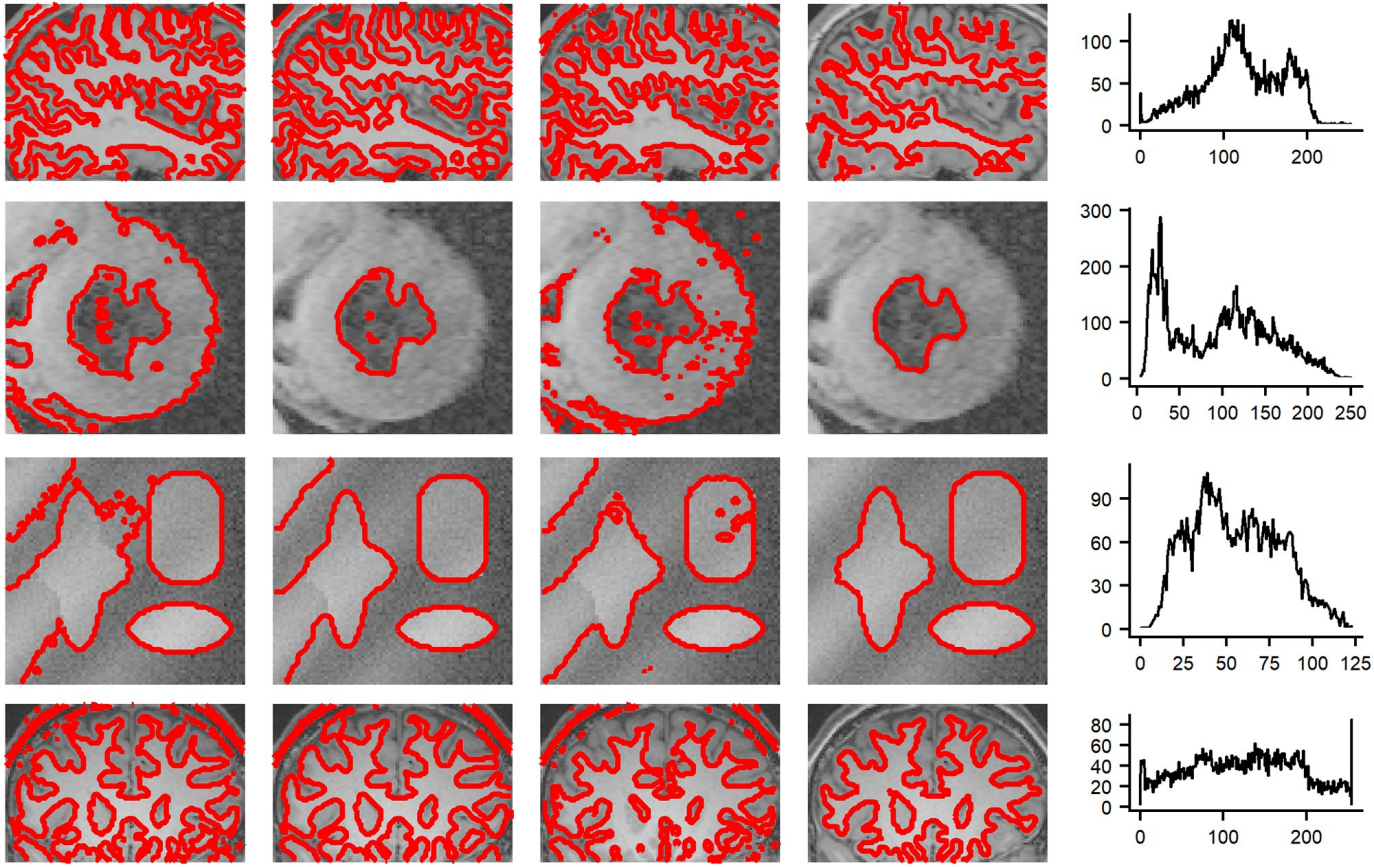
Comparisons with local active contour model using a synthetic image and three medical images with severe intensity inhomogeneity. From left to right: segmentation result by LIC model, LCK model, LGFI model and our method, and the histograms of the image intensity.

We demonstrate the superior performance of the proposed method to previous local active contour methods on a synthetic image (see the third row of Fig 3) and three medical images (The first and last row give two brain images, whereas the second row gives a X-ray image.) with severe intensity inhomogeneity (see the last column of Fig 3). Fig 3 shows the results of the LIC model [12], the LCK model [13] and the LGFI model [14] and our method, all of which consider the local region information and fit them by local intensities means. Therefore, these methods can achieve much better results for images with intensity inhomogeneity than global active contour models. However, The LIC model [12] only uses the local mean information and thereby it fails to segment the objects when the intensity inhomogeneity of images are severe, which can be seen from the first column of Fig 3. Although the LCK model (see the second column of Fig 3) and LGFI model (see the third column of Fig 3) consider the gaussian kernel function as constraint and improve the segmentation results to some extent, these models still fail to discriminate the intensities of the objects. Due to the proper initialization from the coarse segmentation, our method obtain more satisfactory results (see the forth column of Fig 3) than other methods.

Table 2 introduces the computation complexity for Fig 3 in terms of total iterations and CPU time. The proposed method achieves the accurate segmentation results with less CPU time and less number of iterations. The LIC model [12], the LCK model [13] and the LGFI model [14] are all solved by standard gradient method, which take a large number of iterations to converge and make the level set function easily fall into local minima. In order to overcome the drawback of the standard gradient method, our model constructs a steepest descent on the model’s intrinsic manifold which converges extremely fast. Moreover, we split the process of segment into two stage and the second stage can get a suitable initialization from the first stage. This experiment proves our method can obtain the best segmentation results with the least time.

**Table 2:** Iterations and running time (in seconds) for the experiments shown in Fig 3.

Methods		Row1	Row2	Row3	Row4
**LIC**	Iterations	40	50	30	30
	CPU time (s)	3.577	4.081	2.099	2.322
**LCK**	Iterations	100	400	600	200
	CPU time (s)	7.315	29.193	15.929	7.827
**LGFI**	Iterations	100	200	200	100
	CPU time (s)	6.536	15.625	10.343	5.156
**Proposed**	Iterations	**15**	**2**	**10**	**14**
	CPU time (s)	**3.005**	**0.995**	**1.849**	**3.186**

#### Results on natural images with intensity inhomogeneity

**Fig 4:**
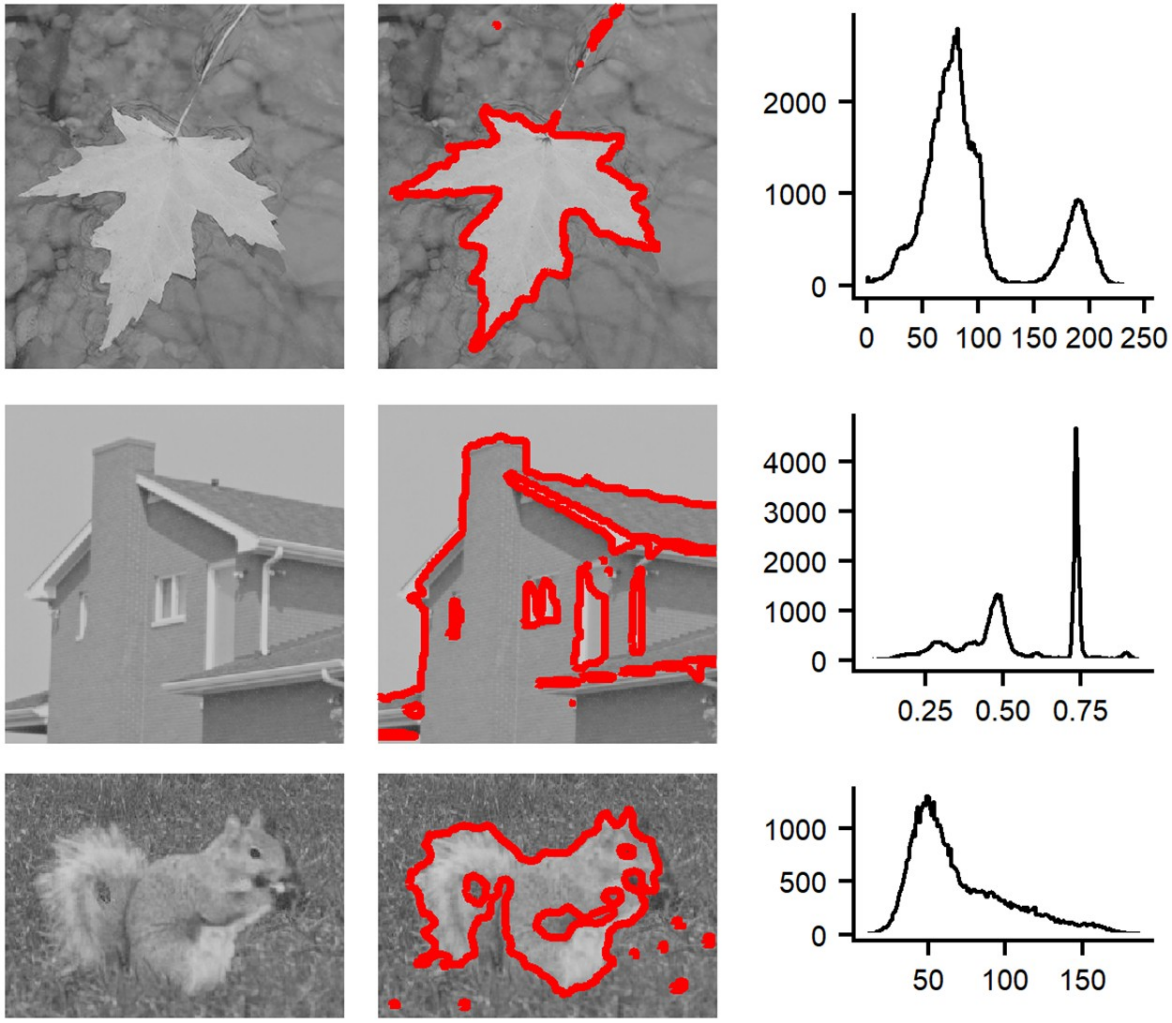
Segmentation results for natural images with intensity inhomogeneity. From left to right: the original images, segmentation results using our methods and the histogram of image intensity.

In Fig 4, we have shown the performance of our method for natural images with different types of region properties. In the third column of Fig 4, the images (see the first column of Fig 4) with severe intensity inhomogeneity see the last column of Fig 4 and some of them with complex textures are used for this experiment. We can see that these images are well segmented into objects and backgrounds from the middle column of Fig 4. This section demonstrates that our method can be applied to handle different types of images.

#### Comparisons with the-state-of-art models on medical images with Poisson noise

**Fig 5:**
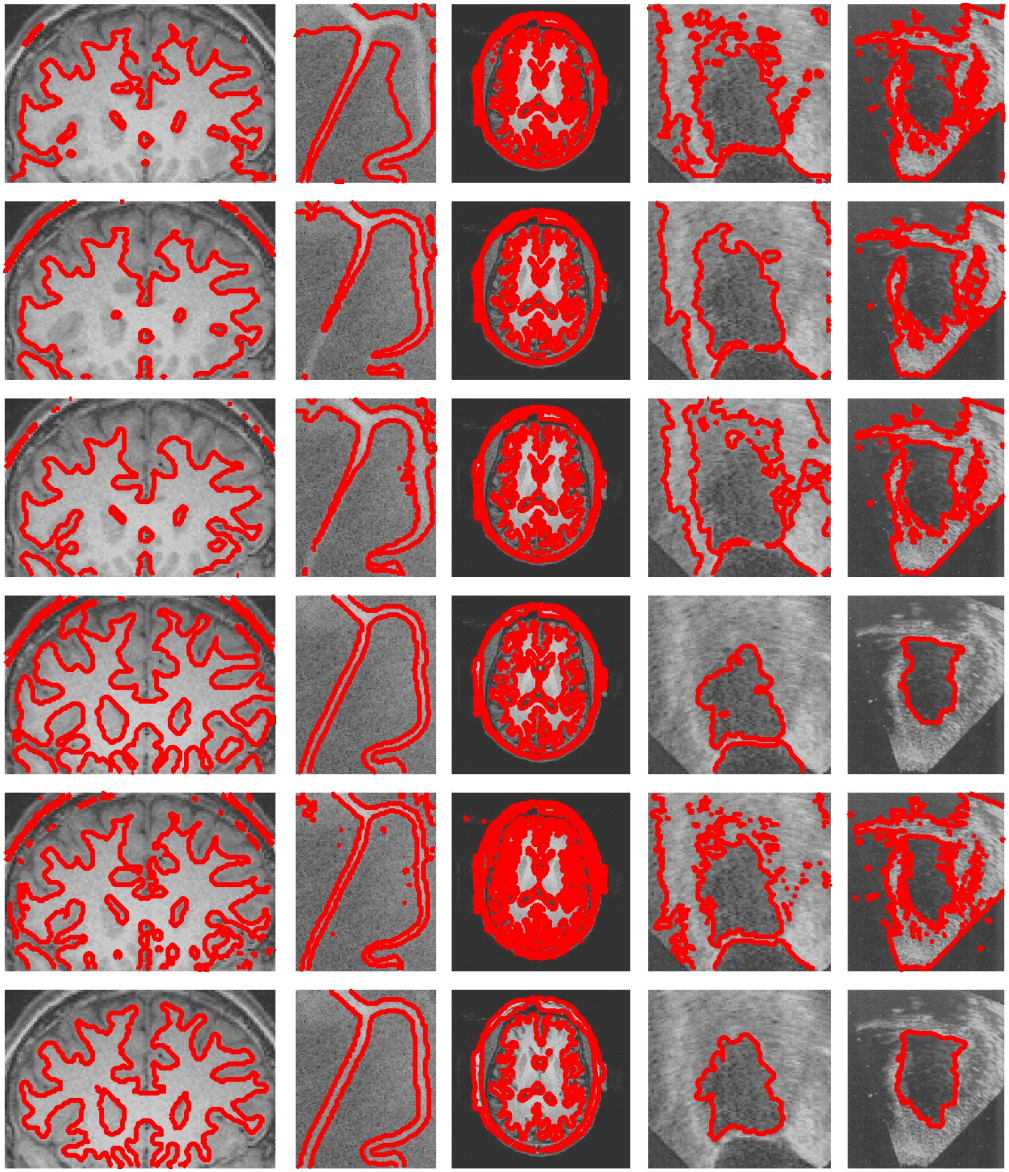
Comparisons with state-of-art methods for niose images. From top to bottom: segmentation results of CV model, GCV model, RCV model, LCK model, LGFI model and our method, respectively.

Fig 5 presents the segmentation results obtained by our method for five medical images (Two brain images are represented in column 1 and column 3, respectively. A blood image is given in column 2. Two ultrasound images are shown in column 4 and column 5.) with Poisson noise, from which we evaluate the performance of proposed method for noise images. The results of CV model, GCV model, RCV model are shown in first, second, third row of the Fig 5, respectively. As can be seen from these segmentation results, the global active contour models can not segment the noise images well. The results of LCK model, LGFI model and the proposed method are shown in forth, fifth and last row of the Fig 5. These local active contour methods all can segment objects from images, whereas the results of the proposed method are clearly better than other local active contour models.

Comparisons of the various methods by the total iterations and CPU time (in seconds) are listed in Table 3, from which we can see that our method converges obviously faster than other methods. On the one hand, our method converges with the Riemannian steepest method is much faster. On the other hand, the first stage provides the proper initialization for second stage. Therefore, our method is also applicable to noise images.

**Table 3:** Iterations and running time (in seconds) for the experiments shown in Fig 5.

Methods		Row1	Row2	Row3	Row4	Row5
**CV**	Iterations	100	200	200	100	100
	CPU time (s)	5.000	11.118	12.018	4.605	5.858
**GCV**	Iterations	100	100	100	100	150
	CPU time (s)	4.451	3.779	4.337	3.203	7.258
**RCV**	Iterations	20	30	20	20	20
	CPU time (s)	3.051	1.953	1.75	1.873	1.825
**LCK**	Iterations	30	500	2000	500	500
	CPU time (s)	2.322	22.510	261.119	77.802	77.280
**LGFI**	Iterations	100	600	600	100	200
	CPU time (s)	5.156	29.234	87.422	4.718	18.078
**Proposed**	Iterations	**14**	**3**	**10**	**12**	**8**
	CPU time (s)	**1.186**	**0.576**	**1.275**	**1.000**	**1.525**

### Quantitative comparisons with the-state-of-art models

To quantitatively analysis the performance of the proposed method, we adopt the **F-score** value [13] and Jaccard similarity (JS) [32] metrics as the evaluation framework of segmentation accuracy. The metric JS is defined as

$$\begin{array}{c} JS=\frac{A⋂B}{A⋃B}, \end{array}$$ (24)

where A is the computed object region and B is the ground truth region. Obviously, the closer the value of JS is to 1, the better segmentation result is obtained. Furthermore, to evaluate the results more precise, we also give the value of **F-score**, which can be calculated by

$$\begin{array}{c} \text{F}-\text{score}=\frac{2\cdot TP}{2\cdot TP+FN+FP}. \end{array}$$ (25)

Where *TP* = *A*⋂*B* (true positive) corresponds the correct segmented regions, FN (false negative) corresponds the false unsegmented regions, FP (false positive) corresponds the undetected segmented regions. Similar to the value of JS, the higher value of **F-score** means the better segmentation results.

#### Robustness to the severe intensity inhomogeneity

**Fig 6:**
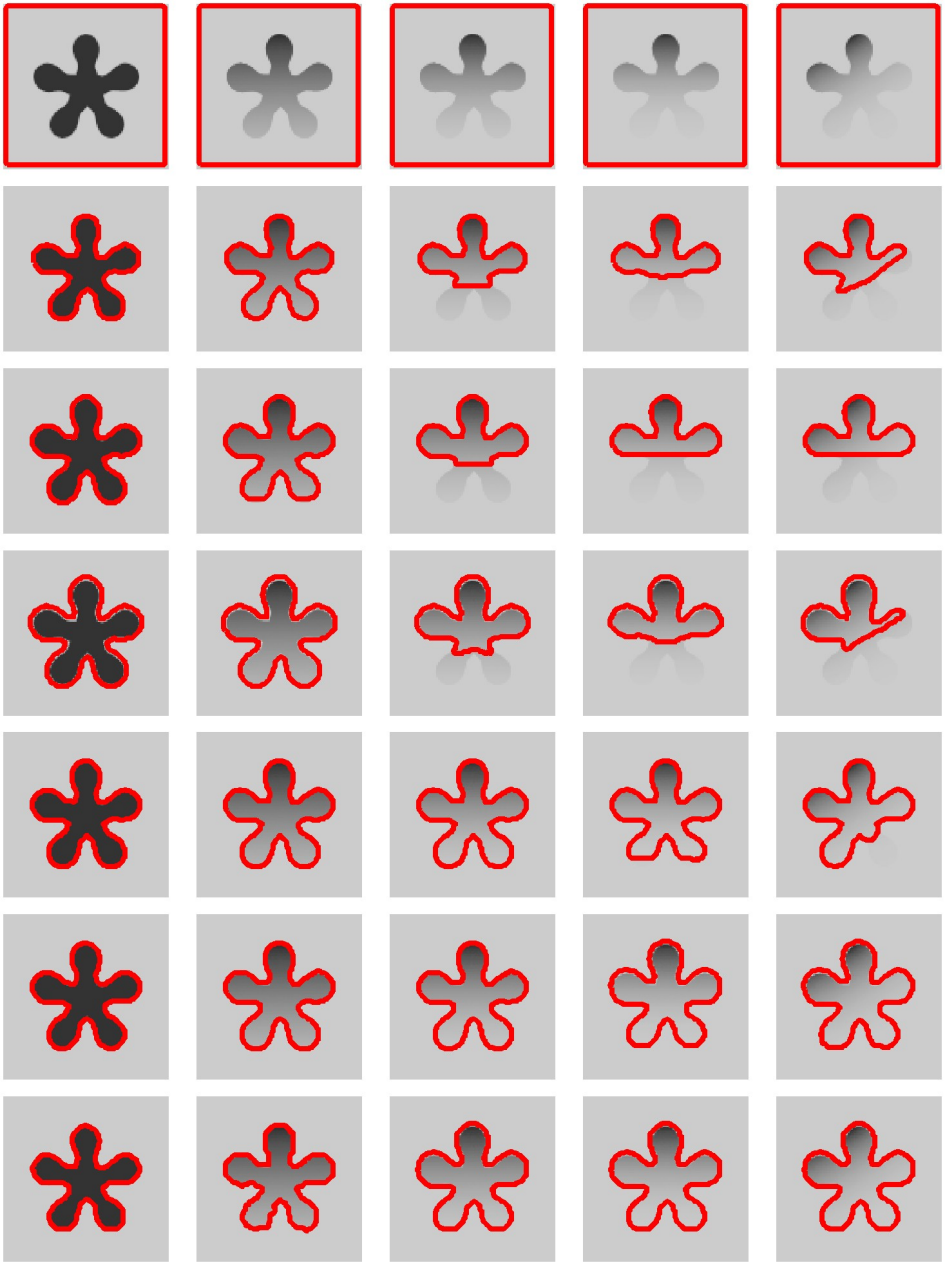
Quantitative comparisons among CV model, GCV model, RCV model, LBF model, LCK model and our method for the images, which the intensity inhomogeneity is gradually increased from left to right.

**Fig 7:**
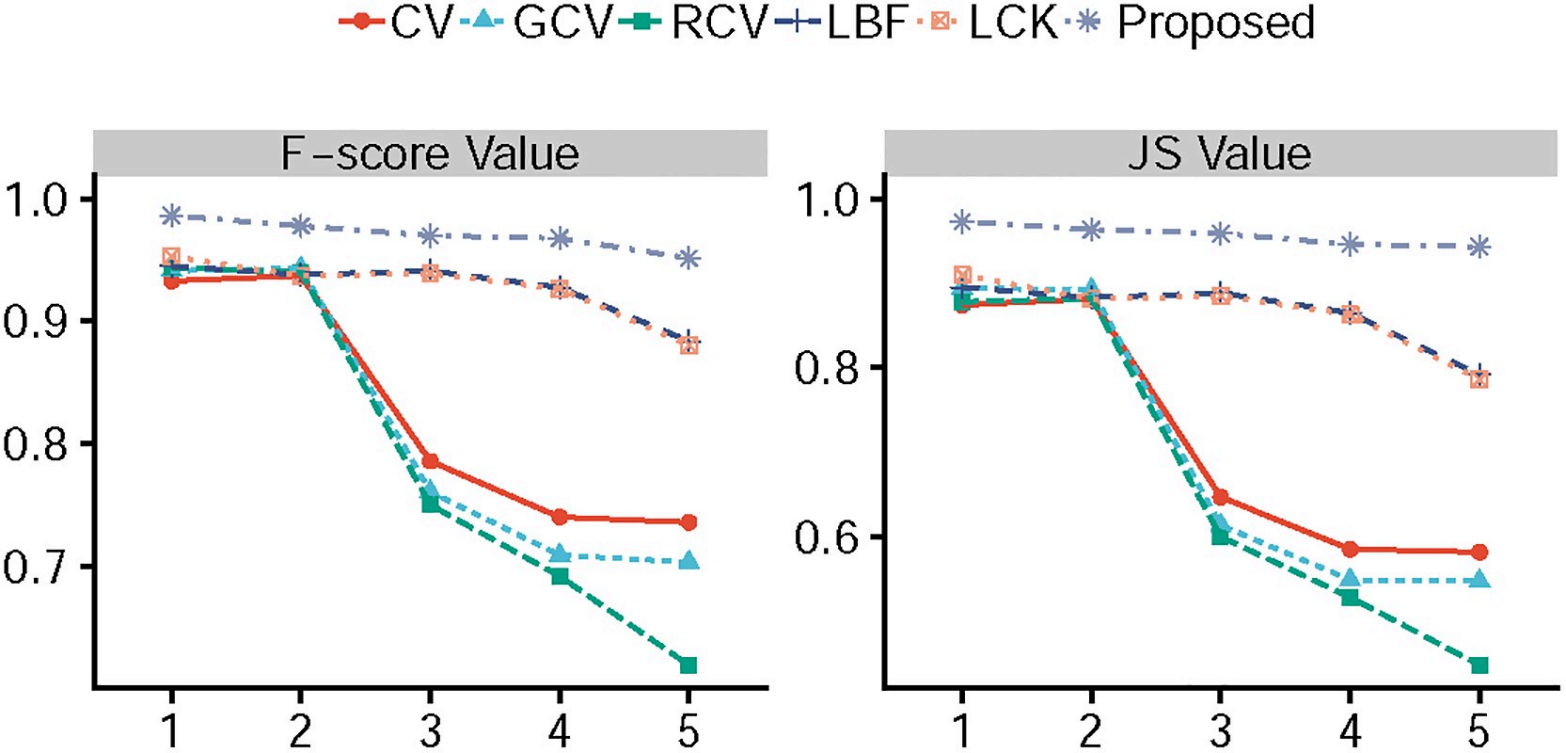
The corresponding F-score value and JS value yields for Fig 6.

Fig 6 shows the results of competing method segmentation on five synthetic images with changed intensity inhomogeneity. The first row of Fig 6 shows the five original images with initial contour. As can be seen, the object of each image is gradual difficult to be segmented from top to bottom. The segmentation results obtained by CV model are presented in the second row, GCV model are presented in the third row, RCV model are presented in the forth row, LBF model are presented in fifth row, LCK model are presented in the sixth row and the proposed model are presented in the last row, respectively. We can see that our method provides the best segmentation results, especially when the strength of the intensity inhomogeneity for objects is strong, which proves that the proposed method is more robust to image intensity inhomogeneity. The corresponding F-score values and JS values are shown in Fig 7, all the models can achieve high F-score values and JS values, when the strength of intensity inhomogeneity is slow (see the first column and second column of Fig 6). Meanwhile, the total iterations and CPU time of all the models, which are shown in Table 4, are close. Whereas, when the strength of intensity inhomogeneity becomes strong, the F-score values and JS values of the proposed method are still close to 1 and higher than other models. Furthermore, our method converges with 2 steps for all images, even the object of image is difficult to be segmented (see the last column of Fig 6). The main difference between our method and other local active contour is the proper initialization and iterative method.

**Table 4:** Iterations and CPU time (in seconds) for the experiments shown in Fig 6.

Methods		Row1	Row2	Row3	Row4	Row5
**CV**	Iterations	5	10	60	60	60
	CPU time (s)	0.847	1.086	3.122	3.035	3.099
**GCV**	Iterations	10	40	60	80	100
	CPU time (s)	0.568	1.987	2.338	3.011	3.254
**RCV**	Iterations	2	10	20	20	15
	CPU time (s)	0.522	0.719	0.908	0.876	0.867
**LBF**	Iterations	200	280	200	400	600
	CPU time (s)	4.192	5.464	4.923	8.814	13.682
**LCK**	Iterations	300	580	640	1400	1300
	CPU time (s)	10.646	22.854	25.619	55.328	55.302
**Proposed**	Iterations	**2**	**2**	**2**	**2**	**2**
	CPU time (s)	**1.142**	**0.180**	**0.299**	**0.325**	**0.394**

#### Robustness to different types of noises

**Fig 8:**
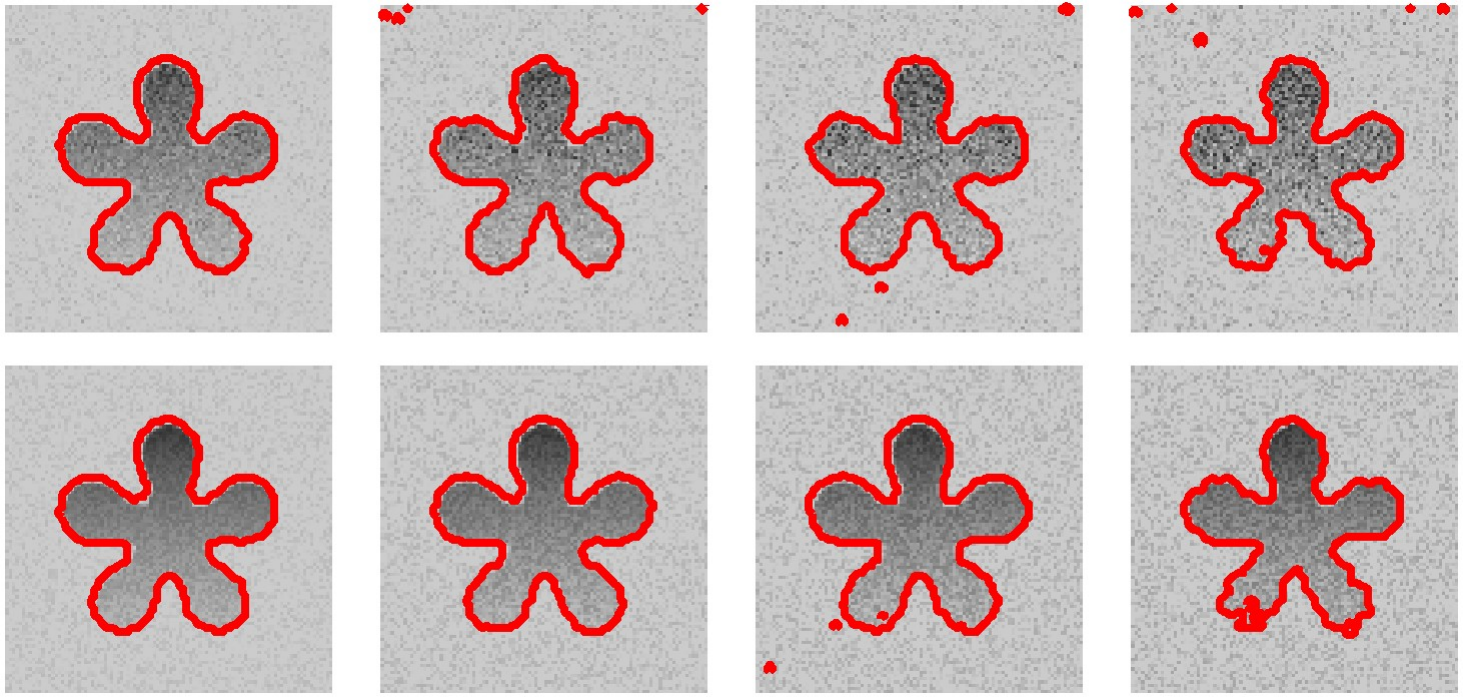
Segmentation results by the proposed method for images with different types of noise. The first row: images with Gaussian white noise (zero means and different variances (*σ* = 0.01, 0.02, 0.03, 0.04)). The second row: images with speckle noise (zeros means and different variances (*σ* = 0.01, 0.02, 0.03, 0.04)).

**Fig 9:**
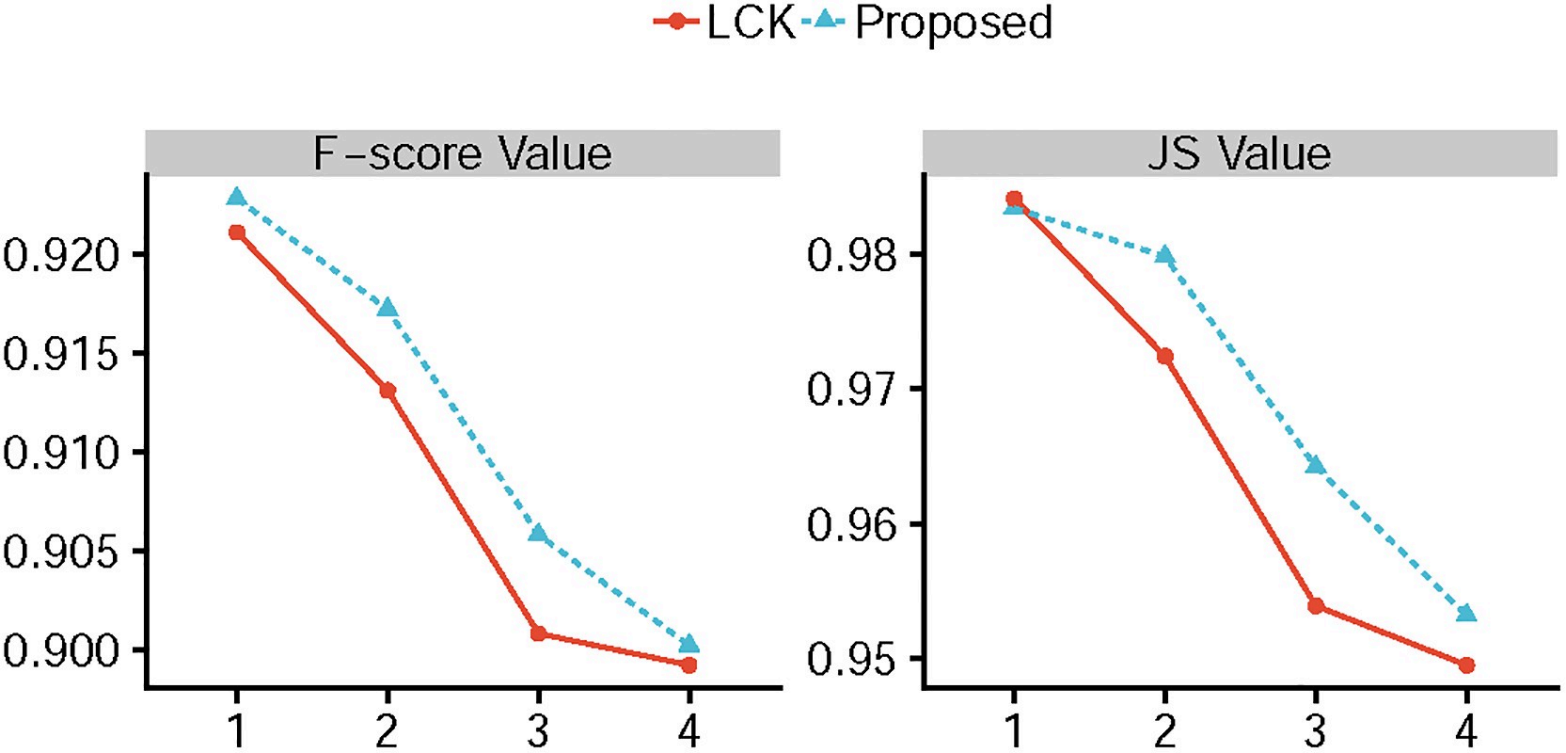
The F-score values and JS values for images shown by Fig 8.

To quantitative illustrate the robustness of our method for different types of noises, we also evaluate the segmentation results by the F-score values and JS values. On the top row of the Fig 8, we show the segmentation results of the proposed method for images with different level of Gaussian noises. And on the bottom row of the Fig 8, we present the segmentation results of the proposed method for images with different level of speckle noise. As can be seen, the proposed method can achieve the satisfactory results even the noise is very strong. Furthermore, the F-score values and JS values for proposed method, which are shown in Fig 9, are also higher than 0.9, so the accuracy of the proposed method is ideal.

## Conclusion

In this paper, we propose a fast two-stage segmentation method for the segmentation problem of synthetic and real-world (including medical) images with complex noise and severe intensity information. The first stage obtains coarse segmentation results in coarse space with low calculation complexity. Following it, the second stage takes the up-sampled contour of the first stage as the initialization and achieves accurate segmentation results in original space quickly. To minimize the energy function with low time complex, we propose a method measuring intensity under the framework of exponential family and converge the model by Riemannian steepest method. Finally, a smooth operator, which is Toeplitz and separable, is used to regularize the level set function to preserve more image details.

The main contribution of this paper is the formulation of two-stage energy function based on LCK model with exponential family for acquiring the intensity information and solving the two-stage method by smooth natural gradient method. Qualitative and quantitative analysis showed that the performance of the proposed method is superior to the LCK model and other state-of-art methods for intensity inhomogeneity images.

There are still some limitations for the proposed method, such as model still depends on the initializations to some extent and model is difficult to segment images with wispy and clutter targets. In the future, we plan to add shape constraints to optimization model and focus on developing the application of the proposed methodology for more types real-life images.

### Appendix

**The computation for** N.

**Theorem 0.1** *Suppose that* Eq (10) *holds. Then the matrix* $N_{\tau }\left(\phi_{\tau }\right))$ *is diagonal with elements*

$$\begin{array}{c} {\left(N_{\tau }\left(\phi_{\tau }\right)\right)}_{\left(x,y\right)}=\{\begin{array}{c} \int_{\Omega_{\tau }}K_{\sigma }\left(x-y\right)\{|\delta \left(\phi \left(y\right)\right)|B_{f}(m_{2}\left(x\right)||m_{1}\left(x\right))\}\;dy\;if\;\phi \left(y\right)\geq 0\\ \int_{\Omega_{\tau }}K_{\sigma }\left(x-y\right)\{|\delta \left(\phi \left(y\right)\right)|B_{f}(m_{1}\left(x\right)||m_{2}\left(x\right))\}\;dy\;if\;\phi \left(y\right)\leq 0 \end{array}, \end{array}$$

*where the K*_*σ*_(*x* − *y*) *is a Gaussian kernel function with width σ, w*_*τy*_ *is the final weight of the y-th pixel. we rearrange the above equation by using convolution method*

$$\begin{array}{c} {\left(N_{\tau }\left(\phi_{\tau }\right)\right)}_{\left(x,y\right)}=K_{\sigma }\left(x\right)*\{\begin{array}{c} \{|\delta \left(\phi_{\tau }\left(x\right)\right)|w_{\tau x}B_{f}(m_{\tau 2}\left(x\right)||m_{\tau 1}\left(x\right))\}\;if\;\phi_{\tau }\left(x\right)\geq 0\\ \{|\delta \left(\phi_{\tau }\left(x\right)\right)|w_{\tau x}B_{f}(m_{\tau 1}\left(x\right)||m_{\tau 2}\left(x\right))\}\;if\;\phi_{\tau }\left(x\right)\leq 0 \end{array}, \end{array}$$ (26)

*as i* = *j and* ${\left(N_{\tau }\left(\phi_{\tau }\right)\right)}_{\left(x,y\right)}=0$ *otherwise, the B*_*f*_ (m*||*m*) *is the f* − *Bregman divergence*

$$\begin{array}{c} B_{f}(m^{*}\left||m\right)≜A\left(m^{*}\right)-A\left(m\right)-\left\langle \eta \left(m^{*}\right)-\eta \left(m\right),\nabla_{\eta }A\left(m\right)\right\rangle , \end{array}$$ (27)

*In above equations, A*(⋅) *denotes the logarithm of the normalising constant of f and* ∇_*η*_ *A*(*m*) *is the gradient of A*(⋅) *with respect to the canonical constant vector η* = *η*(*θ*). *Furthermore, δ*′(*u*) *is evaluated by a regularised approximation* $\delta_{\epsilon }^{{}^{\prime }}\left(u\right)=\frac{-2\epsilon }{\pi }sign\left(u\right)\max\left(u,\epsilon \right)/{\left(\epsilon^{2}+u^{2}\right)}^{2}$, *which is bounded away from zero, then matrix* $N_{\tau }\left(\phi_{\tau }\right)$ *is positive definite*.

**Proof**. To prove these results we first give the definition of the Fisher information matrix (FIM) [33]

$$\begin{array}{c} {\left(G\left(\phi \right)\right)}_{\left(i,j\right)}≜-E_{I|\phi }\left\{\frac{\partial^{2}}{\partial \phi_{i}\partial \phi_{j}}\;\text{log}\;\left[f\left(I|\phi \right)\right]\right\}, \end{array}$$ (28)

where *E*_*I*|*ϕ*_ represents the expectation operator of the function *f*(*I*(*x*)|*ϕ*) and *f*(*I*(*x*)|*ϕ*(*x*)) = ∫_{*x*:*ϕ*(*x*)>0}_ *f*(*I*(*x*)|*m*_1_(*x*)) d*x* + ∫_{*x*:*ϕ*(*x*)<0}_ *f*(*I*(*x*)|*m*_2_(*x*)) d*x*. Following above definition, we develop the derivatives of Fisher information matrix (FIM)

$$\begin{array}{ccc} {\left(G\left(\phi \right)\right)}_{\left(x,y\right)}= & & -\delta \left(\phi \left(y\right)\right)E[\;\text{log}\;f\left(I\left(y\right)|m_{1}\left(x\right)\right)|\phi \left(y\right)]\\ & & -\delta \left(-\phi \left(y\right)\right)E[\;\text{log}\;f\left(I\left(y\right)|m_{2}\left(x\right)\right)|\phi \left(y\right)] \end{array}$$ (29)

and then the $N_{\tau }\left(\phi_{\tau }\right)$ is

$$\begin{array}{ccc} {\left(N_{\tau }\left(\phi_{\tau }\right)\right)}_{\left(x,y\right)}= & & K_{\sigma }\left(x\right)*\{-\delta \left(\phi_{\tau }\left(x\right)\right)w_{\tau x}E\left[\;\text{log}\;f\left(I\left(x\right)|m_{\tau 1}\left(x\right)\right)|\phi_{\tau }\left(x\right)\right]\\ & & -\delta \left(-\phi_{\tau }\left(x\right)\right)w_{\tau x}E\left[\;\text{log}\;f\left(I\left(x\right)|m_{\tau 2}\left(x\right)\right)|\phi_{\tau }\left(x\right)\right]\}. \end{array}$$ (30)

if *x* = *y* and (*G*(*ϕ*))_(*x*,*y*)_ = 0, then $N_{\tau }\left(\phi_{\tau }\right)=0$. Otherwise, we need to prove the diagonal elements of matrix $N_{\tau }\left(\phi_{\tau }\right)$ are strictly positive.

In Eq (30), the *E*(…|*ϕ*_*τ*_(*x*)) is the expectation with respect to the marginal likelihood

$$\begin{array}{c} f\left(I\left(y\right);\phi \left(x\right)\right)=\{\begin{array}{c} f\left(I\left(y\right);m_{\tau 1}\left(x\right)\right)\;if\;\phi_{\tau }\left(x\right)\geq 0\\ f\left(I\left(y\right);m_{\tau 2}\left(x\right)\right)\;if\;\phi_{\tau }\left(x\right)<0. \end{array} \end{array}$$ (31)

According to the fact that *δ*′(−*x*) = −*δ*′(*x*) and the Eq (30) can be rewritten as

$$\begin{array}{c} {\left(N\left(\phi_{\tau }\right)\right)}_{\left(x,y\right)}=K_{\sigma }\left(x\right)*\left\{-\delta^{{}^{\prime }}\left(\phi_{\tau }\left(x\right)\right)w_{\tau x}E\left[\;\text{log}\;\left(\frac{f(I\left(x\right)|m_{\tau 1}\left(x\right))}{f(I\left(x\right)|m_{\tau 2}\left(x\right))}\right)\right]|\phi_{\tau }\left(x\right)\right\}. \end{array}$$ (32)

In terms of Kullback-Leibler divergences [34], we obtain

$$\begin{array}{c} {\left(N_{\tau }\left(\phi_{\tau }\right)\right)}_{\left(x,y\right)}=K_{\sigma }\left(x\right)*\{\begin{array}{c} \left\{\right|\delta^{{}^{\prime }}\phi_{\tau }\left(x\right)w_{\tau x}|KL_{f}(m_{\tau 1}\left(x\right)||m_{\tau 2}\left(x\right))\}\;if\;\phi_{\tau }\left(x\right)\geq 0\\ \left\{\right|\delta^{{}^{\prime }}\phi_{\tau }\left(x\right)w_{\tau x}|KL_{f}(m_{\tau 2}\left(x\right)||m_{\tau 1}\left(x\right))\}\;if\;\phi_{\tau }\left(x\right)<0 \end{array}, \end{array}$$ (33)

In above equation, the Kullback-Leibler divergence is

$$KL(m_{\tau 1}\left(x\right)||m_{\tau 2}\left(x\right))≜\int_{{ℜ}^{p}}\;\text{log}\left(\frac{f\left(s;m_{\tau 1}\left(x\right)\right)}{f\left(s;m_{\tau 2}\left(x\right)\right)}\right)f\left(s;m_{\tau 1}\left(x\right)\right)\;\text{d}s.$$

Finally, in this paper the function *f* belongs to exponential family and we can rewritten (33) in terms of Bregman divergences [34]

$${\left(N_{\tau }\left(\phi_{\tau }\right)\right)}_{\left(x,y\right)}=K_{\sigma }\left(x\right)\{\begin{array}{lll} \left\{|\delta \prime \phi_{\tau }\left(x\right)w_{\tau x}|B_{f}(m_{\tau 1}\left(x\right)||m_{\tau 2}\left(x\right))\right\} & if & \phi_{\tau }\left(x\right)\geq 0\\ \left\{|\delta \prime \phi_{\tau }\left(x\right)w_{\tau x}|B_{f}(m_{\tau 2}\left(x\right)||m_{\tau 1}\left(x\right))\right\} & if & \phi_{\tau }\left(x\right)<0 \end{array},$$

Due to the fact that the terms *B*_*f*_(*m*_*τ*1_(*x*)||*m*_*τ*2_(*x*))} and *B*_*f*_(*m*_*τ*2_(*x*)||*m*_*τ*1_(*x*))} are strictly positive, and then the diagonal elements of matrix $N_{\tau }\left(\phi_{\tau }\right)$ are strictly positive.

## Supporting information

S1 Dataset Contains synthetic images, real-world images and medical images used in Figs 1, 2, 3, 4, 5, 6 and 8. (ZIP)

S1 Supporting Information Contains MATLAB code of the proposed method. (ZIP)
