# Supplementary figures and images for: A fast two-stage active contour model for intensity inhomogeneous image segmentation
Source: PLoS One. 2019 Apr 19;14(4):e0214851. doi: 10.1371/journal.pone.0214851 (PMC6474649; doi:10.1371/journal.pone.0214851)

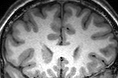

Supplement: S1 Dataset — (ZIP) [file pone.0214851.s001.zip › S1 Dataset/1.bmp]

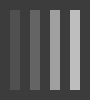

Supplement: S1 Dataset — (ZIP) [file pone.0214851.s001.zip › S1 Dataset/12.bmp]

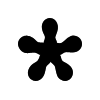

Supplement: S1 Dataset — (ZIP) [file pone.0214851.s001.zip › S1 Dataset/16.bmp]

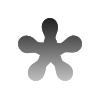

Supplement: S1 Dataset — (ZIP) [file pone.0214851.s001.zip › S1 Dataset/17.bmp]

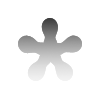

Supplement: S1 Dataset — (ZIP) [file pone.0214851.s001.zip › S1 Dataset/18.bmp]

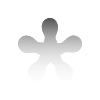

Supplement: S1 Dataset — (ZIP) [file pone.0214851.s001.zip › S1 Dataset/19.bmp]

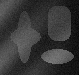

Supplement: S1 Dataset — (ZIP) [file pone.0214851.s001.zip › S1 Dataset/2.bmp]

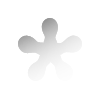

Supplement: S1 Dataset — (ZIP) [file pone.0214851.s001.zip › S1 Dataset/20.bmp]

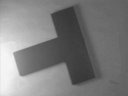

Supplement: S1 Dataset — (ZIP) [file pone.0214851.s001.zip › S1 Dataset/3.bmp]

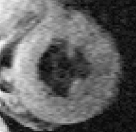

Supplement: S1 Dataset — (ZIP) [file pone.0214851.s001.zip › S1 Dataset/41.bmp]

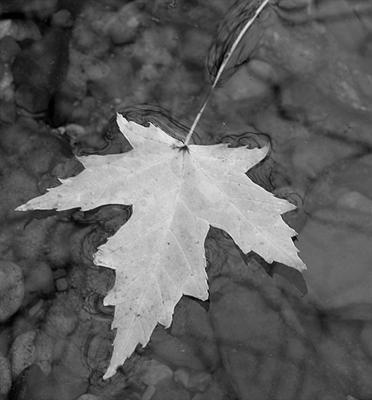

Supplement: S1 Dataset — (ZIP) [file pone.0214851.s001.zip › S1 Dataset/42.bmp]

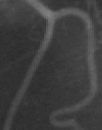

Supplement: S1 Dataset — (ZIP) [file pone.0214851.s001.zip › S1 Dataset/5.bmp]

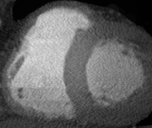

Supplement: S1 Dataset — (ZIP) [file pone.0214851.s001.zip › S1 Dataset/7.bmp]

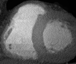

Supplement: S1 Dataset — (ZIP) [file pone.0214851.s001.zip › S1 Dataset/7D.bmp]

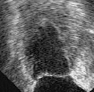

Supplement: S1 Dataset — (ZIP) [file pone.0214851.s001.zip › S1 Dataset/h.bmp]

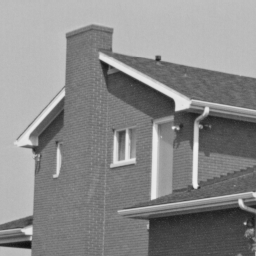

Supplement: S1 Dataset — (ZIP) [file pone.0214851.s001.zip › S1 Dataset/house.png]

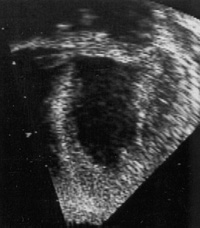

Supplement: S1 Dataset — (ZIP) [file pone.0214851.s001.zip › S1 Dataset/lv.bmp]

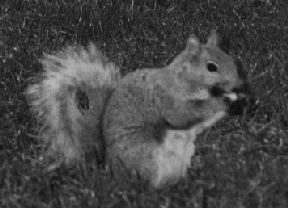

Supplement: S1 Dataset — (ZIP) [file pone.0214851.s001.zip › S1 Dataset/squirrel.bmp]

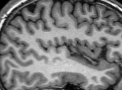

Supplement: S1 Dataset — (ZIP) [file pone.0214851.s001.zip › S1 Dataset/T2.bmp]
